# Lower Extremity Osteoarthritis: A Risk Factor for Mental Health Disorders, Prolonged Opioid Use, and Increased Resource Utilization After Single-Level Lumbar Spinal Fusion

**DOI:** 10.5435/JAAOSGlobal-D-21-00280

**Published:** 2022-03-17

**Authors:** Justin J. Turcotte, Paul J. King, Chad M. Patton

**Affiliations:** From the Department of Orthopedics, Luminis Health Anne Arundel Medical Center, Annapolis, MD.

## Abstract

Few studies have examined the effect of hip or knee osteoarthritis, together described as lower extremity osteoarthritis (LEOA) on patient outcomes after lumbar fusion. The purpose of this study was to evaluate the effect of LEOA on postoperative outcomes and resource utilization in patients undergoing single-level lumbar fusion.

**Methods:**

Using a national deidentified database, TriNetX, a retrospective observational study of 17,289 patients undergoing single-level lumbar fusion with or without a history of LEOA before September 1, 2019, was conducted. The no-LEOA and LEOA groups were propensity score matched, and 2-year outcomes were compared using univariate statistical analysis.

**Results:**

After propensity score matching, 2289 patients with no differences in demographics or comorbidities remained in each group. No differences in the rate of repeat lumbar surgery were observed between groups (all *P* > 0.30). In comparison with patients with no LEOA, patients with LEOA experienced higher rates of overall and new onset depression or anxiety, prolonged opioid use, hospitalizations, emergency department visits, and ambulatory visits over the 2-year postoperative period (all *P* < 0.02).

**Conclusion:**

Patients with LEOA undergoing single-level lumbar fusion surgery are at higher risk for suboptimal outcomes and increased resource utilization postoperatively. This complex population may benefit from additional individualized education and multidisciplinary management.

As the global population ages, the burden of musculoskeletal disease and associated use of surgical intervention are expected to continue increasing.^1^ From 2004 to 2015, the volume of elective lumbar fusions increased 62.3%, with the largest increase among patients aged 65 years or older.^2^ In parallel, the prevalence of hip and knee osteoarthritis (OA)—which has been identified as one of the leading causes of global disability—is expected to increase 49% to 78.4 million or 25.9% of the US adults by 2040.^3,4^ In a 2012 study, 32.5% of the patients older than 50 years undergoing spine surgery were identified as having concomitant pathological hip changes.^5^ Based on these trends, increasing prevalence of lower extremity OA in patients undergoing lumbar fusion is expected, warranting the evaluation of surgical outcomes in this complex population.

Normal spine-pelvis-lower extremity alignment is critical to maintaining an ergonomic standing posture, and pathology of any element of the chain can lead to poor balance and pain.^6^ The relationship between the spine, hips, and pelvis in maintaining sagittal alignment and balance have been well described.^7,8^ Pathology in either the lumbopelvic complex or hip joint can clinically affect the other and is defined as spine-hip syndrome or hip-spine syndrome, depending on whether the primary source of pathology lies in the spine or hip, respectively.^9^ In the presence of hip OA, hip mobility and flexion is inhibited, causing the lumbopelvic complex to compensate through increased lordosis, which may lead to spinal degeneration and the associated pain and complications of hyperlordosis.^9^ Although less commonly studied, knee OA also influences alignment of the spine-pelvis-lower extremity axis. In patients with severe knee OA, the lumbar spine has been identified as the primary mechanism of compensation to maintain alignment, with hip flexion and pelvic anteversion providing additional compensation.^10^

Many previous studies have evaluated whether lumbar spinal stenosis or lumbar fusion negatively affects the outcomes of total joint arthroplasty.^11^ Most of these studies evaluate the relationship between total hip arthroplasty (THA) and lumbar fusion. Patients undergoing THA followed by lumbar fusion have been shown to have higher complication rates, revision surgery rates, postoperative pain scores, and narcotic usage than those not undergoing subsequent fusion.^12,13^ Similarly, patients undergoing THA after lumbar fusion have been shown to have decreased satisfaction, less improvement in overall pain, and worse quality of life than those without previous fusion.^13,14^ Direct comparison of these two groups has shown that patients undergoing THA before lumbar fusion are at an increased risk of postoperative dislocation, infection, revision surgery, and prolonged opioid use compared with those undergoing THA after lumbar fusion.^15^ By contrast, fewer studies have evaluated whether lower extremity arthritis affects the results of lumbar spine surgery. This relatively limited evidence suggests that decreased ambulatory status (a common clinical presentation of lower extremity OA) is associated with decreased postoperative function, walking ability, and satisfaction after the surgical treatment of lumbar spinal stenosis, but that overall improvement in a variety of patient-reported outcome (PRO) measures is similar in patients undergoing lumbar fusion with or without lower extremity OA.^11^ The purpose of this study was to evaluate the effect of lower extremity OA on postoperative outcomes and resource utilization in patients undergoing single-level lumbar fusion.

## Methods

This study was deemed institutional review board exempt as a review of deidentified aggregated data by the institutional clinical research committee. The TriNetX Research Network database was queried as of October 4, 2021. Patient cohorts and outcome measures were defined using Current Procedural Terminology (CPT) and International Classification for Disease 10th edition (ICD-10) diagnosis codes. Patients undergoing single-level lumbar fusion before September 1, 2019, were included to allow for 2 years of the postoperative follow-up. All patients in both cohorts underwent CPT 22612 (single-level posterior or posterolateral arthrodesis) or CPT 22630 (single-level posterior interbody arthrodesis) with no additional multilevel CPT codes (22614 or 22632). Patients in the hip or knee OA cohort (referred to as lower extremity OA [LEOA]) had a diagnosis of hip OA (M16) or knee OA (M17) documented before undergoing lumbar fusion, whereas patients in the no hip or knee OA cohort (referred to as no LEOA) did not have either of these diagnosis codes documented before surgery. A total of 17,289 patients (LEOA n = 2483 and no LEOA n = 14,806) were included in this study.

The two cohorts were then propensity score matched on age, race, sex, diagnoses of overweight or obese, anxiety disorder, or major depressive disorder. Outcomes were then assessed over the 2-year postoperative period. These included repeat lumbar spine surgery, total hip or knee arthroplasty, diagnoses of depression or anxiety, newly diagnosed depression or anxiety not present before surgery, and prolonged opioid use. Prolonged opioid use was defined as the documented prescription of any opioid medication after 1 year postoperatively. Resource utilization measures included hospitalizations, emergency department (ED) visits, and ambulatory visits. For these measures, both the incidence rates and number of occurrences were compared. Hospitalizations were defined as any inpatient admission or observation status hospital encounters in ≥30 days after surgery to avoid double counting any prolonged hospitalizations for the index fusion procedure. Categorical outcomes were compared between cohorts using z-tests, and the risk ratio and 95% confidence interval (CI) were calculated. Continuous outcome measures were compared using two-sided independent sample Student *t*-tests. Statistical significance was assessed at α = 0.05. All statistical analyses were conducted within the TriNetX platform.

### About TriNetX

TriNetX is a “global health research network that optimizes clinical research and enables discoveries through the generation of real-world evidence.”^16^ The research platform includes longitudinal data from 56 healthcare organizations and includes more than 78 million patients. As a federated network, TriNetX received a waiver from Western institutional review board because only aggregated counts, statistical summaries of deidentified information, but no protected health information are received, and no study-specific activities are done in retrospective analyses. Deidentified, Health Insurance Portability and Accountability Act (HIPAA) compliant electronic health record data are collected from participating healthcare organizations who submit structured and unstructured data elements. Variables captured include demographics, diagnoses (all mapped to ICD-10 coding), procedures (ICD-10 procedure coding system (PCS) and CPT), medications, laboratory values, and genomic information. Statistical analysis is conducted within the analytics platform using parallel R and Python queries triangulated to maximize test accuracy.^17^

## Results

**Figure 1:**
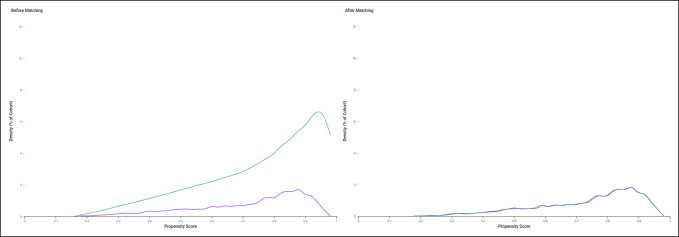
Line graph showing propensity score–matching density function. Green line denotes patients without hip or knee osteoarthritis. Purple line denotes patients with hip or knee osteoarthritis.

Before propensity score matching, patients with lower extremity OA were statistically older, more likely to be female, and had higher rates of overweight or obesity, anxiety disorders, and major depressive disorders (all *P* < 0.01). No statistically significant difference in the proportion of White patients was observed (*P* = 0.92). After propensity score matching, 2289 patients were included in each group; no statistically significant differences in any of the demographics or comorbidities remained between groups (all *P* > 0.05) (Table 1). A graphical depiction of the propensity score and density score functions before and after matching is presented in Figure 1.

**Table 1:** Patient Demographics and Characteristics Before and After Propensity Score Matching

Demographic or Characteristic, n (%)	Before Matching			After Matching		
	Hip or Knee OA (n = 2483)	No Hip or Knee OA (n = 14,806)	*P*-Value	Hip or Knee OA (n = 2289)	No Hip or Knee OA (n = 2289)	*P*-Value
Age (yr – avg. ± SD)^a^	64.8 ± 10.8	55.9 ± 15.3	**<0.01**	64.5 ± 10.8	64.4 ± 10.9	0.91
White race	1962 (82.6)	11406 (82.7)	0.92	1889 (82.5)	1885 (82.4)	0.88
Female	1440 (60.6)	7579 (54.9)	**<0.01**	1378 (60.2)	1392 (60.8)	0.67
Overweight or obese	951 (40.0)	1816 (13.2)	**<0.01**	867 (37.9)	894 (39.1)	0.41
Anxiety disorder	607 (25.5)	1659 (12.0)	**<0.01**	548 (23.9)	542 (23.7)	0.84
Major depressive disorder	166 (7.0)	334 (2.4)	**<0.01**	133 (5.8)	104 (4.5)	0.05

*P* values < 0.05 are in bold.

a Denotes two-sided independent samples Student *t*-test.

The outcomes evaluated over the 2-year postoperative period were grouped into surgical interventions, mental health disorders and opioid use, and resource utilization. No statistically significant differences in rates of repeat lumbar spine surgery were observed between groups (LEOA: 5% versus no LEOA: 5%, *P* = 0.69). Similarly, no statistically significant differences in rates of lumbar fusion (LEOA: 4% versus no LEOA: 4%, *P* = 0.82) or lumbar decompression (LEOA: 3% versus no LEOA: 2%, *P* = 0.34) were observed. Patients with a prior diagnosis of LEOA were more likely to undergo primary THA or total knee arthroplasty after lumbar fusion (LEOA: 8% versus no LEOA: 1%, Risk Ratio (RR) = 6.586 [95% CI: 4.476 to 9.692], *P* < 0.01) (Table 2).

**Table 2:** 2-Year Postoperative Outcomes

Outcome Measure	Hip or Knee OA Patients With Outcome (n, %)	No Hip or Knee OA Patients With Outcome (n, %)	RR (OA: No OA)	RR 95% CI	*P*-Value
Surgical interventions					
Repeat lumbar spine surgery (decompression or fusion)	122 (5.3)	116 (5.1)	1.052	0.821-1.347	0.69
Lumbar fusion	96 (4.2)	93 (4.1)	1.032	0.781-1.365	0.82
Lumbar decompression	62 (2.7)	52 (2.3)	1.192	0.829-1.716	0.34
Primary THA or TKA	191 (8.3)	29 (1.3)	6.586	4.476-9.692	**<0.01**
Mental health disorders and opioid use					
Depression or anxiety	757 (33.1)	628 (27.4)	1.205	1.103-1.317	**<0.01**
New onset depression or anxiety	156 (11.7)	121 (8.8)	1.339	1.069-1.677	**0.01**
Prolonged opioid use	1090 (47.6)	836 (36.5)	1.304	1.217-1.397	**<0.01**
Resource utilization					
Hospitalization	788 (34.4)	595 (26.0)	1.324	1.211-1.448	**<0.01**
No. of hospitalizations^a^	2.0 ± 7.9	1.2 ± 4.1	N/A	N/A	**<0.01**
ED visit	700 (30.6)	483 (21.1)	1.449	1.311-1.602	**<0.01**
No. of ED visits (avg. ± SD)^a^	0.9 ± 2.3	0.6 ± 2.2	N/A	N/A	**<0.01**
Ambulatory visit	2060 (90.0)	2007 (87.7)	1.026	1.006-1.048	**0.01**
No. of ambulatory visits (avg. ± SD)^a^	48.7 ± 61.5	29.9 ± 46.0	N/A	N/A	**<0.01**

CI = confidence interval, ED = emergency department, OA = osteoarthritis, RR = risk ratio, THA = total hip arthroplasty, TKA = total knee arthroplasty

*P* values <0.05 are in bold.

a Denotes two-sided independent samples Student *t*-test.

The evaluation of mental health disorders and opioid use demonstrated that patients with LEOA were at an increased risk for depression or anxiety, new onset depression or anxiety, and prolonged opioid use after lumbar fusion (all *P* < 0.05). The relative risk in the LEOA group was 1.205 (95% CI: 1.103 to 1.317, *P* < 0.01) for depression or anxiety, 1.339 (95% CI: 1.069 to 1.677, *P* = 0.01) for new onset depression or anxiety, and 1.304 (95% CI: 1.217 to 1.397, *P* < 0.01) for prolonged opioid use (Table 2).

Across all measures of resource utilization—hospitalizations, ED visits, and ambulatory visits—patients in the LEOA group were more likely to use these services postoperatively (all *P* < 0.01). Thirty-four percent of the patients with LEOA were hospitalized, compared with 26% of the patients with no LEOA (RR: 1.324, 95% CI: 1.211 to 1.448, *P* < 0.01). Furthermore, the LEOA group experienced a higher number of hospitalizations (LEOA: 2 ± 8 versus no LEOA: 1 ± 4, *P* < 0.01). Similarly, 31% of the patients with LEOA had an ED visit, compared with 21% of the patients with no LEOA (RR = 1.449, 95% CI: 1.311 to 1.602, *P* < 0.01) and patients with LEOA required more ED visits (LEOA: 1 ± 2 versus no LEOA: 1 ± 2, *P* < 0.01). As expected, most patients in both groups had postoperative ambulatory visits, although patients with LEOA were significantly more likely to seek ambulatory care (LEOA: 90% versus no LEOA: 88%, *P* < 0.01). More notably, patients in the LEOA group required a higher number of ambulatory visits than those without LEOA (LEOA: 49 ± 61 versus no LEOA: 30 ± 46, *P* < 0.01) (Table 2).

## Discussion

This study demonstrates that patients with degenerative lower extremity arthritis undergoing single-level lumbar fusion surgery are at a higher risk for ongoing depression or anxiety, new onset depression or anxiety, prolonged opioid use, and increased resource utilization in the two-year postoperative period. These results build on previous studies showing that this population may be at risk for complications and poorer patient-reported outcomes after lumbar spine surgery. Based on these results, we suggest that this population may benefit from additional individualized education and management and that enhanced multidisciplinary management of this complex population may improve outcomes.

To date, a relatively few studies evaluating the relationship between lower extremity OA and outcomes of lumbar spine surgery exist.^11^ A recent study by Djurasovic et al.^11^ evaluated the topic and concluded that patients with lower extremity arthritis who undergo lumbar fusion can achieve meaningful improvements in patient reported outcomes (PROs) similar to patients without arthritis. Using propensity score–matched cohorts of 110 patients undergoing lumbar fusion with or without concomitant lower extremity OA, the study found similar improvements in back pain, leg pain, Oswestry Disability Index, and EuroQol 5 Dimensions of Health scores between groups 12 months postoperatively. Similarly, Eneqvist et al. compared PROs between 440 matched patients undergoing lumbar surgery with or without a history of THA and found no difference in EuroQol 5 Dimensions of Health, EQ visual analog scale, leg pain, Oswestry Disability Index, or satisfaction at the 1-year follow-up. However, a history of THA was associated with worse postoperative back pain visual analog scale (β = 5.3, 95% CI: 0.3 to 10.3), leading the suggestion that communication of the potential for continued back pain is important to communicate and set appropriate expectations in this population.^18^ In a systematic review of 21 studies, Aalto et al.^19^ evaluated preoperative predictors of clinical outcomes after surgery for lumbar spinal stenosis, and identified depression, cardiovascular comorbidity, disorders influencing walking ability, and scoliosis as predictors of poorer subjective outcomes. Although hip and knee OA was not specifically evaluated, its undisputed effect on the walking ability has been cited as the basis for considering this as a relevant finding when considering the importance of lower extremity OA in this population.^11^ Taken together, these studies describe mixed findings regarding the effect of lower extremity OA on PROs and subjective outcomes after spine surgery. This study's finding that patients with degenerative lower extremity OA underwent similar rates of repeat lumbar spine surgery within 2 years postoperatively suggests that the previously described differences in PROs and subjective outcomes might not be suitable enough for patients to elect to undergo additional surgical intervention. However, this study only evaluated single-level lumbar fusions specifically, and the heterogeneity in surgical indications, procedures done, and outcomes evaluated across studies limit any broad conclusions that may be drawn.

Depression, anxiety, and chronic opioid use are relatively common in the complex spine population.^20,21^ Although surgery has been frequently demonstrated to improve overall quality of life and reduce reliance on narcotics,^22,23,24,25^ a subset of patients will unfortunately experience exacerbation or new onset of mental health disorders or rely on opioids for a prolonged period of time postoperatively.^25-27^ Early identification of patients at risk for these outcomes is critical to provide access to resources and formulate strategies for mitigating the risk. Preoperative opioid use, preoperative depression and anxiety, smoking, Black race, more levels fused have been previously identified as risk factors for postoperative depression and anxiety and prolonged opioid use after spine surgery.^21,28-30^ Based on the results of this study, patients with concomitant lower extremity arthritis are at 20% to nearly 34% increased relative risk of postoperative depression, anxiety, and prolonged opioid use, suggesting that this must be considered as a notable risk factor for postoperative mental health disorders and chronic opioid use.

Given the increasing prevalence of spine surgery and its high cost,^2,31^ it is critical that interventions aimed at improving the value of care delivered be developed and evaluated. Postoperative utilization of resources, such as skilled nursing facilities, ED visits, and readmissions, have been well described to increase the cost of care after spine surgery.^32-34^ The first step toward improving the value is identifying populations at risk for increased utilization of these resources. In a previous study at our institution,^35^ patients with increased comorbidity burden were found to have higher rates of 30-day readmissions after lumbar fusion, in alignment with multiple other studies.^36,37^ Beyond overall comorbidity burden, increased age, female sex, preoperative levels of pain and function, socioeconomic factors, and a variety of individual comorbidities, including depression and anxiety, diabetes, congestive heart failure, chronic obstructive pulmonary disease, and obesity, have all been linked with increased resource utilization and cost of care after lumbar fusion.^38-40^ This study suggests that lower extremity OA is also at risk for increased postoperative care requirements after lumbar fusion because these patients experienced higher rates of hospitalization, ED visits, and ambulatory visits and required markedly more of all three types of visits than the matched cohort without lower extremity OA.

In light of these findings, we must ask the question “How can care processes be redesigned to mitigate the potentially negative implications of lower extremity OA on the outcomes and value of lumbar fusion?” Although the answer to this question remains elusive, we suggest two synergistic steps be considered: (a) increased nurse navigation and education tailored to this population and (b) increased integration of the historically fragmented joint and spine care teams. Across both the total joint and spine populations, multiple studies have highlighted the positive effect of preoperative education and nurse navigation on postoperative outcomes and the value of surgery.^41,42,43,44^ At our institution, recently published data found that patients attending nurse navigator–led preoperative education courses experienced shorter lengths of stay, decreased cost of care, and decreased postacute resource utilization in the total joint and lumbar fusion populations.^45,46^ Given the potential for suboptimal outcomes in patients with lower extremity OA undergoing lumbar fusion, we suggest all these patients be managed through a nurse navigator. Furthermore, although nurse navigation currently focuses on optimizing patients during the perioperative period, a paradigm shift toward using navigators longitudinally may improve patient engagement and enhance access to appropriate specialists in patients suffering from multiple musculoskeletal diseases. Finally, we suggest that integration of joint and spine programs to manage patients with both spine and lower extremity conditions is warranted. Establishment of a multidisciplinary conference to discuss treatment strategies and identify patients who may benefit from consultation with outside specialists, such as pain management or mental health providers, is a first step toward improving outcomes in this complex population. Similar to the concept of multidisciplinary tumor boards, which have been repeatedly demonstrated to improve outcomes in cancer care,^47^ this model holds promise to improve coordination across the increasingly subspecialized field of orthopaedics.

This study does have multiple limitations. First, its use of a national deidentified database relies heavily on coded data, which has been shown to have notable limitations including potentially variable fidelity.^48,49^ Second, although the database incorporates a national sample of institutions, it is possible that patients included in this study are not representative of the broader population of patients undergoing single-level lumbar fusion. Third, we did not stratify our population by the various indications for surgery. Given the heterogeneity of the lumbar fusion population, it is possible that the presence of lower extremity OA disproportionately affected the outcomes of specific diagnoses. Fourth, we were unable to further subdivide our population into isolated hip or knee OA based on database sample size requirements to prevent potential patient reidentification. This leaves an important area of opportunity into future research regarding whether differences in the type of lower extremity OA differentially affect outcomes after spinal fusion. Fifth, it is likely that some unknown and uncontrolled for confounding factors influence our results. Although the cohorts in this study were propensity score matched on baseline demographics and mental health status, we did not account for the multitude of comorbidities that can influence outcomes and postoperative utilization. This approach was intentionally selected to reduce overfitting and capture the largest possible representative sample of patients undergoing single-level lumbar fusion but does increase the potential effect of confounding comorbidities. One specific example of an important confounding factor that was not controlled for was preoperative opioid use, which may have affected and skewed our results. Finally, the differences in patient characteristics and outcomes presented in this study were assessed based on statistical significance and may not be of clinical significance in practice. Despite these limitations, we suggest this study holds value as the first large national evaluation of the effect of lower extremity OA on surgical intervention, mental health status, opioid use, and resource utilization after lumbar fusion.

## Conclusion

Patients with degenerative lower extremity arthritis undergoing single-level lumbar fusion surgery are at higher risk for ongoing depression or anxiety, new onset depression or anxiety, prolonged opioid use, and increased resource utilization in the 2-year postoperative period. This population may benefit from additional individualized education and management, and enhanced multidisciplinary management of this complex population may improve outcomes.
